# In vivo injection of *α*‐bungarotoxin to improve the efficiency of motor endplate labeling

**DOI:** 10.1002/brb3.468

**Published:** 2016-04-27

**Authors:** Wentao Chen, Tingting Yu, Bo Chen, Yisong Qi, Peixun Zhang, Dan Zhu, Xiaofeng Yin, Baoguo Jiang

**Affiliations:** ^1^ Department of Trauma and OrthopaedicsPeking University People's HospitalNo. 11 South Xizhimen StreetBeijing100044China; ^2^ Britton Chance Center for Biomedical PhotonicsWuhan National Laboratory for OptoelectronicsHuazhong University of Science and Technology1037 Luoyu RoadWuhanHubei430074China

**Keywords:** Distribution, in vivo injection, motor endplate, optical clearing technique, *α*‐bungarotoxin

## Abstract

**Introduction:**

Motor endplates are composed of a motor neuron terminal and muscle fiber and are distributed in skeletal muscle, causing muscle contraction. However, traditional motor endplate staining methods are limited to the observation of partial skeletal muscle. The procedure was time‐consuming due to strict incubation conditions, and usually provided unsatisfactory results. We explored a novel method to label motor endplate rapidly by in vivo injection of fluorescent *α*‐bungarotoxin.

**Methods:**

Fifty‐two mice were randomly divided into two groups, an experiment group (*n* = 50), and a contrast group (*n* = 2). In experiment group, *α*‐bungarotoxin was injected via the caudal vein. The injection dosages were designated as 0.1, 0.2, 0.3, 0.4, and 0.5 *μ*g/g. The experimental mice were divided into five subgroups of ten mice per group. The contrast group was only injected with 200 *μ*L normal saline solution. Bilateral gastrocnemius were acquired for microscope analysis and optical clearing to seek specific fluorescent signal.

**Results:**

A dose of 0.3 *μ*g/g of *α*‐bungarotoxin with 1 h conjugation time could display the number and structure of motor endplate in plane view. Compared with the traditional procedure, this method was rapid, convenient, and time‐saving. Combined with the optical clearing technique, spatial distribution could also be seen, helping to better understand the stereoscopic view of motor endplate position in skeletal muscle.

**Conclusions:**

In vivo injection of *α*‐bungarotoxin proved effective for studying motor endplate in skeletal muscle.

## Introduction

MEP (Motor endplate) was described in 1928 by Cajal (Sanes and Lichtman 1999). MEP receives motor neuron transmission signals to maintain muscle tone. Degeneration of MEP caused by nerve trauma and autoimmune antibodies leads to muscle dysfunction and atrophy. Research into nervous system development (Wu et al. 2010), myasthenia gravis (Lindstrom 2000), and nerve injury repair (Kang et al. 2014) has focused on the MEP. The *α*‐BTX (*α*‐bungarotoxin) conjugated with fluorescent dyes or radioisotopes clearly reveals the structure of MEP, and has been applied in research to enable morphological observation (Sine 1997; Wu and Mei 2013). However, traditional MEP staining methods that are time‐consuming, lead to indistinct staining and are limited to the partial local characteristic observation of skeletal muscle. MEPs can be viewed under fluorescent microscope in limited areas representing only a portion of muscle. The full observation of MEP in skeletal muscle requires a large amount of tissue sections, which is time‐consuming and complicated. Based on the characteristics of *α*‐BTX that include high affinity and irreversible binding to MEP, we hypothesized that *α*‐BTX conjugated with fluorescent dye could be injected in vivo to label the MEP.

## Materials and Methods

### Animals

This study was performed in accordance with the recommendations of the Institutional Animal Care Guidelines and approved ethically by the Administration Committee of Experimental Animals, Peking University People's Hospital (Beijing, China, Permit number: 2013‐24).

A total of 52 mice with a homogeneous C57BL/6 background, aged 8‐weeks, and each weighing 20 ± 2 g, were obtained from the Laboratory Animal Center of Peking University (Beijing, China) and anesthetized with 10% chloral hydrate via intraperitoneal injection. The animals were randomly divided into two groups, an experiment group (*n* = 50), and a contrast group (*n* = 2). In the experiment group, Alexa Fluor 594‐Bungarotoxin (B13423; Invitrogen, New York, NY) was injected via the caudal vein. The injection dosages were designated as 0.1, 0.2, 0.3, 0.4, and 0.5 *μ*g/g. The experimental mice were divided into five subgroups of ten mice per group. The *α*‐BTX was diluted into 200 *μ*L distilled water. The contrast group was injected with 200 *μ*L normal saline solution.

### Histology

The mice were over anesthetized to death 1, 2, 4, 6, and 8 h after the *α*‐BTX injection and tissues samples were immediately taken. Animals were perfused transcardially with 0.9% sodium chloride solution, then with 4% paraformaldehyde solution. The intact bilateral gastrocnemius was dissected from the proximal femoral attachment and distal Achilles tendon, with surrounding tissues left intact, then postfixed with 4% paraformaldehyde for 12 h at 4°C. The samples were dehydrated in 20% and 30% sucrose for 8 h. Consecutive frozen sections measuring 60 *μ*m were taken using a freezing microtome (Leica CM1900, Leica Microsystems, Heidelberg, Germany). Slides were stored at −20°C before analysis. Twelve slides from the proximal, middle, and distal portion of gastrocnemius muscle were selected for analysis under confocal microscope for positive fluorescent signal.

### Confocal microscope analysis

The sections were viewed under confocal microscope (TCS SP8‐Confocal‐MP‐FLIM; Leica) with 561 nm Argon/Krypton laser line exciting the Alexa Fluor. MEP images were collected with a 10× microscope objective. Once a fluorescent signal was positive in the microscopic field, 60‐*μ*m thick stacks in *Z*‐axis (2 *μ*m steps) were acquired, as the area of interest was confirmed, with a 40× oil immersion microscope objective. Each optical section was then added to a stack of images using Leica data processing software (LAS AF, Leica, Heidelberg, Germany).

### Optical clearing technique

The method of optical clearing was based on the 3DISCO technique (Erturk et al. 2012). The dissected and postfixed gastrocnemius muscle was treated with serial incubation in 50%, 70%, 80%, and 100% tetrahydrofuran for full dehydration and further incubation in 100% dibenzyl ether for refractive index matching. During the clearing procedure, the sample was placed on a rotator for gentle shaking for 2 h. The fluorescence images were acquired with Ultramicroscope (LaVisionBioTec, Bielefeld, Germany), equipped with MV PLAPO 2×/0.5 dry objective (W.D. 20 mm), and dipping extension cap. The acquired images were analyzed using Image J software (NIH, Bethesda, MD) and reconstructed into MIP (maximum‐intensity projection).

## Results

**Figure 1:**
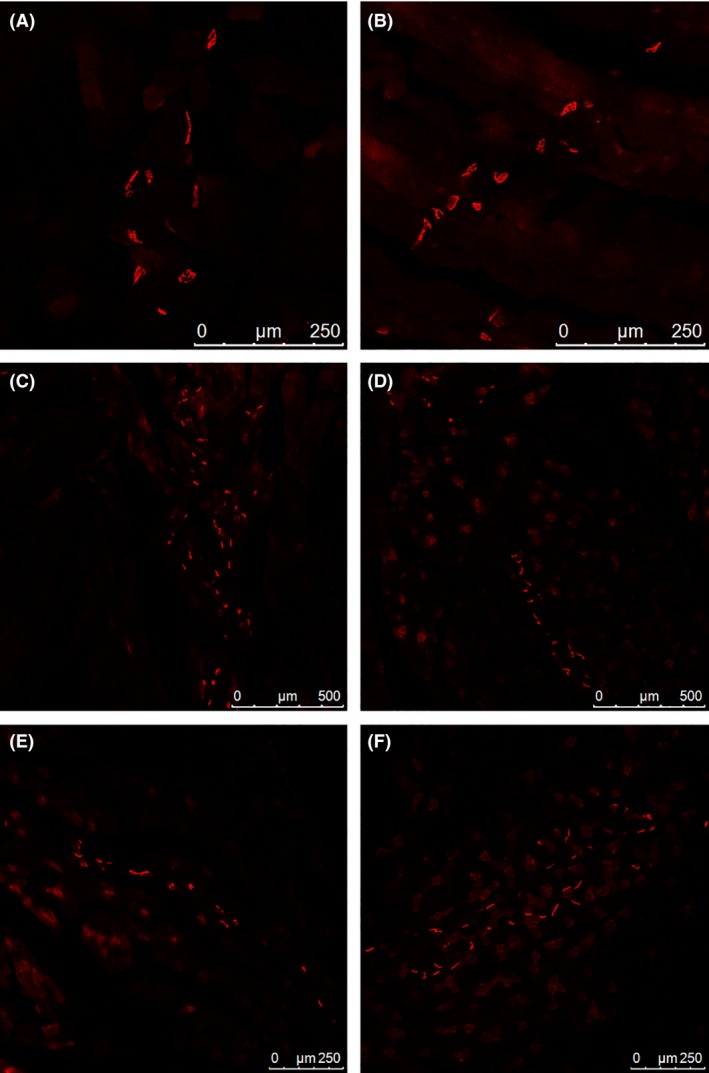
Fluorescent images of different subgroups under confocal microscopy. Significant and specific fluorescent signal from motor endplate (light red point) as viewed in the 0.3 *μ*g/g subgroup, and autofluorescence of gastrocnemius found in the background of the section (A). The specific fluorescent signal also found in the 0.5 *μ*g/g subgroup (B). A fluorescent signal visible under microscopy 1 h after 0.3 *μ*g/g dosage injection (C), remaining stable at 2 h (D), 4 h (E), and 8 h (F) after injection.

**Figure 2:**
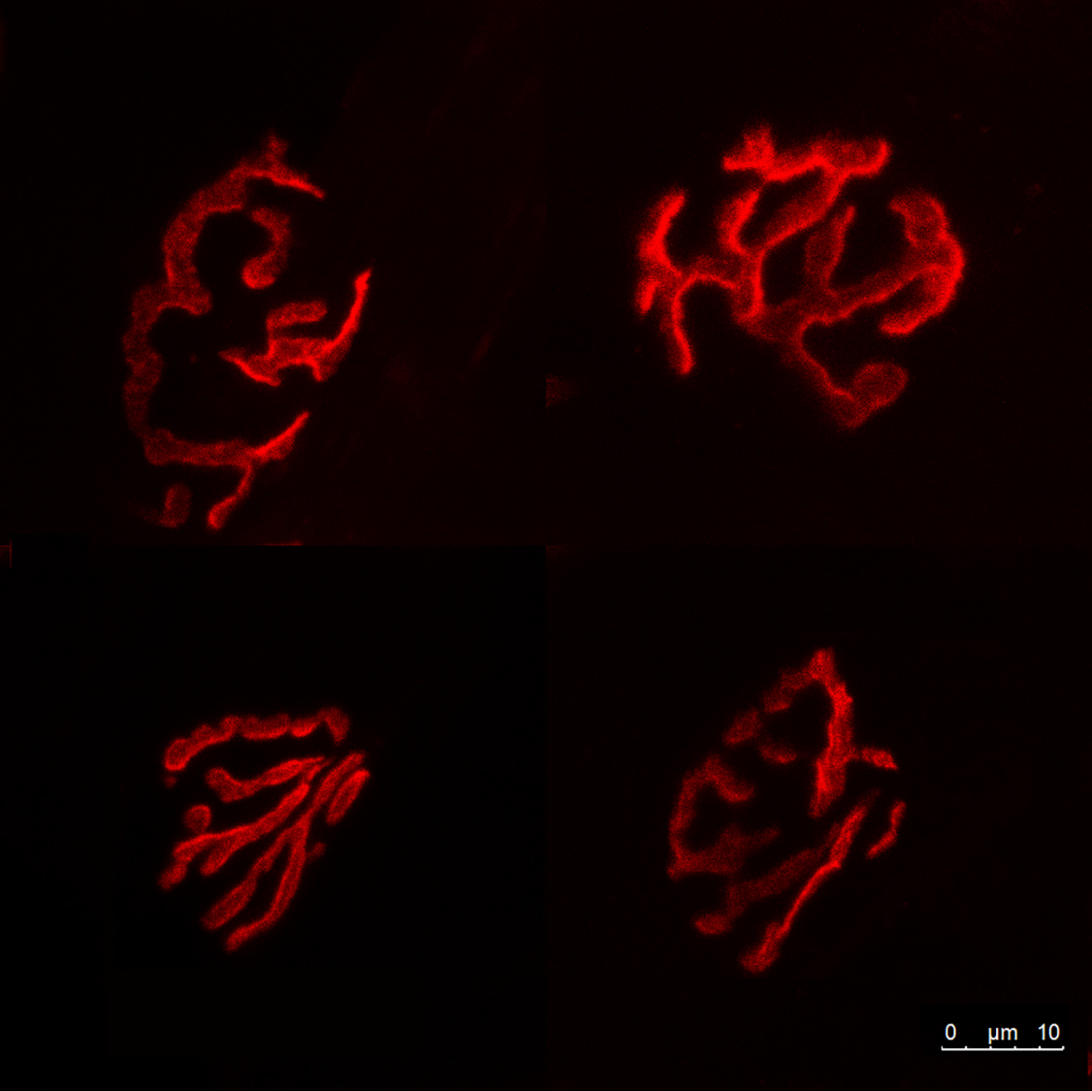
Flat plane projection of Z‐stacked confocal image of gastrocnemius muscle. The structure of motor endplate appears “pretzel‐shaped”, revealing different types of morphology.

**Figure 3:**
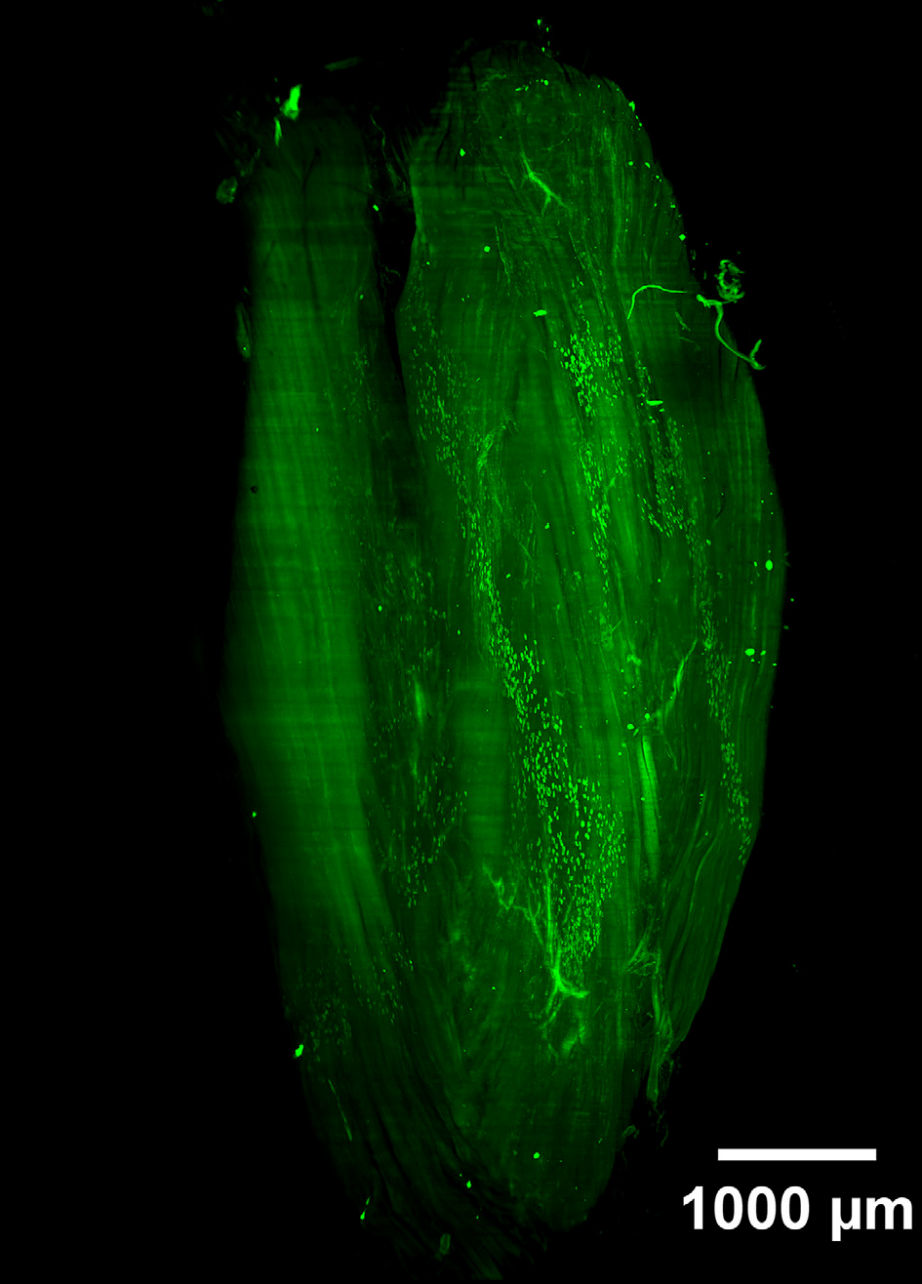
The distribution of motor endplate (MEP) in gastrocnemius was revealed by optical clearing technique. The light green points represented MEP, distributed in “root‐shaped” clusters. Image is a maximum‐intensity projection of Z stacks (thickness = 1.5 mm). The upper section represents medial and lateral heads of gastrocnemius, and lower section, Achilles tendon.

Under confocal microscope, the specific fluorescent signal (bright red) of MEP could be viewed in the 0.3 *μ*g/g subgroup (Fig. 1A and B) and stabilized after more than 1 h injection of *α*‐BTX (Fig. 1C–F & Table 1). MEP flat plane projection of the Z‐stacked confocal image in the gastrocnemius showed “pretzel‐shaped” appearance (Fig. 2). MIP revealed that MEP in gastrocnemius was distributed in linear clusters, in a “root” shape (Fig. 3).

**Table 1:** The positive fluorescent signal found in different dosage and time subgroups

Time (h)/Dosage (*μ*g/g)	0.1	0.2	0.3	0.4	0.5
1	−	−	+	+	+
2	−	−	+	+	+
4	−	−	+	+	+
6	−	−	+	+	+
8	−	−	+	+	+

“+:”Specific fluorescent signal of MEP viewed under confocal microscope.

“−:” Autofluorescence of gastrocnemius found with nonspecific fluorescent signal.

## Discussion

Motor endplate plays an important role in muscle contraction and damage to MEP has been considered to contribute to functional deficits after nerve injury (Pratt et al. 2013). Previous studies have revealed that MEP is not permanent (Fambrough et al. 1979), and can change and remodel during functional recovery (Tomas i Ferre et al. 1987). More recently, *α*‐BTX has been applied for observation of the morphological structure of MEP. Traditional staining methods were performed with tissue sections taken, followed by incubation with *α*‐BTX, but this is time‐consuming and complicated. With the help of confocal fluorescent microscopy, a laser can penetrate 500 *μ*m sections that approximately equal the thickness of small flat muscles (Hama et al. 2011). The whole‐mounting technique reveals MEP distribution in small and flat muscle tissue, such as intracranial muscle (Murray et al. 2010), diaphragm, and sternocleidomastoid (Lichtman et al. 1987; Marques and Santo Neto 2002). However, the whole‐mounting technique did not work for larger muscles such as gastrocnemius. For further observation of internal MEP in larger skeletal muscles, consecutive sections of muscle are needed, potentially causing loss of information and misjudgment of MEP position. Therefore, other techniques have been explored for investigation of MEP distribution in larger muscles.

The molecular weight of *α*‐BTX is 8000 Da. It shows high affinity and specific binding to MEP. Based on these characteristics, we first injected *α*‐BTX in vivo. In one previous study, ^125^I‐ BTX was injected into tibialis anterior muscles. The results showed a rapid loss of radioactive signal within the first minutes of injection, indicating that intramuscularly injected ^125^I‐BTX did not effectively bind to MEP (Strack et al. 2011). Therefore, we performed intravenous *α*‐BTX injection through the caudal vein in anaesthetized mice. According to known lethal dosages to humans and to mice, the dosages used were 0.1, 0.2, 0.3, 0.4, and 0.5 *μ*g/g, to explore effective and minimal dosage. In the experimental group, mice were divided into several subgroups. At different time points after injection, intact gastrocnemius was acquired for further histological examination.

The results showed that *α*‐BTX could penetrate vessel walls and bind the MEP located in gastrocnemius. The structure of MEP was clearly visible with dosages of more than 0.3 *μ*g/g (Fig. 1A and B). One hour was enough to label MEP with stable fluorescent signal after injection (Fig. 1C–F & Table 1). The MEP image for the gastrocnemius muscle was “pretzel” like (Fig. 2), as described in previous study (Pratt et al. 2013). These results revealed that a 0.3 *μ*g/g dosage and 1 h conjugation time was sufficient for the visualization of MEP. Compared with traditional procedures, it was a rapid and convenient method of labeling MEP, facilitating MEP staining.

Optical clearing technique can facilitate internal and deep microstructure imaging in tissue with preservation of tissue integrity, and are widely used in medical imaging for accurate reconstruction (Yu et al., 2011; Hama et al. 2015). Using a combination of in vivo injection of *α*‐BTX and the optical clearing technique enabled us to explore MEP distribution in gastrocnemius in our research. During the optical clearing procedure, the fluorescent Alexa Fluor was stable with no significant fluorescent signal attenuation under confocal microscopy. The maximum intensity projection of MEP in gastrocnemius revealed that MEP was distributed in linear clusters, arranged in a “root” shape (Fig. 3). This may correspond with Christensen's findings in a different perspective (Christensen 1959) and also proves that injected *α*‐BTX can bind the internal MEP in skeletal muscle.

Our novel technique is proposed as a method of obtaining accurate information about MEP in skeletal muscle, with no loss of detail of the integral structure. It can also be extrapolated that integrated MEP information from other skeletal muscles could be acquired, such as from the anterior tibialis, quadriceps femoris, and biceps brachii, after only one intravenous injection of *α*‐BTX. This information can be applied to the study of MEP in myasthenia gravis, nervous system development, and degeneration and regeneration after nerve injury, to obtain information such as number and structure.

In conclusion, a 0.3 *μ*g/g dose of *α*‐BTX with 1 h conjugation time results in clear display of the MEP in C57BL/6 mice. This novel technique for studying MEP in skeletal muscle simplifies the traditional immunohistochemistry procedure and is rapid, convenient, and effective.

## Conflict of Interest

The authors declare no conflict of interest.

## Data Availability Statement

The data described in our manuscript are fully available through FigShare. They were divided into several rar files and uploaded separately. The list of DOI's includes https://dx.doi.org/10.6084/m9.figshare.2998975.v1, https://dx.doi.org/10.6084/m9.figshare.3046666.v1, https://dx.doi.org/10.6084/m9.figshare.3048088.v1, https://dx.doi.org/10.6084/m9.figshare.3048343.v1, https://dx.doi.org/10.6084/m9.figshare.3048649.v1, https://dx.doi.org/10.6084/m9.figshare.3049225.v1, https://dx.doi.org/10.6084/m9.figshare.3049687.v1, https://dx.doi.org/10.6084/m9.figshare.3049843.v1, https://dx.doi.org/10.6084/m9.figshare.3050017.v1, https://dx.doi.org/10.6084/m9.figshare.3050851.v1, https://dx.doi.org/10.6084/m9.figshare.3051319.v1, https://dx.doi.org/10.6084/m9.figshare.3051481.v1, https://dx.doi.org/10.6084/m9.figshare.3051697.v1, https://dx.doi.org/10.6084/m9.figshare.3052057.v1, https://dx.doi.org/10.6084/m9.figshare.3052294.v1, https://dx.doi.org/10.6084/m9.figshare.3052591.v1, https://dx.doi.org/10.6084/m9.figshare.3052969.v1.
